# Superior Place Learning of C57BL/6 vs. DBA/2 Mice Following Prior Cued Learning in the Water Maze Depends on Prefrontal Cortical Subregions

**DOI:** 10.3389/fnbeh.2019.00011

**Published:** 2019-01-29

**Authors:** Woo-Hyun Cho, Jung-Cheol Park, Won Kyung Jeon, Jeiwon Cho, Jung-Soo Han

**Affiliations:** ^1^Department of Biological Sciences, Konkuk University, Seoul, South Korea; ^2^Herbal Medicine Research Division, Korea Institute of Oriental Medicine (KIOM), Daejeon, South Korea; ^3^Convergence Research Center for Diagnosis, Treatment and Care System of Dementia, Korea Institute of Science and Technology (KIST), Seoul, South Korea; ^4^Department of Medical Science, College of Medicine, Catholic Kwandong University International St. Mary’s Hospital, Incheon, South Korea; ^5^Institute for Bio-Medical Convergence, Incheon St. Mary’s Hospital, The Catholic University of Korea, Incheon, South Korea

**Keywords:** task switching, CREB phosphorylation, place learning, cued learning, medial prefrontal cortex, orbitofrontal cortex, water maze

## Abstract

The participation of the prefrontal cortex (PFC), hippocampus, and dorsal striatum in switching the learning task from cued to place learning were examined in C57BL/6 and DBA/2 mice, by assessing changed levels of phosphorylated CREB (pCREB). Mice of both strains first received cued training in a water maze for 4 days (4 trials per day), and were then assigned to one of four groups, one with no place training, and three with different durations of place training (2, 4, or 8 days). Both strains showed equal performance in cued training. After the switch to place training, C57BL/6 mice with 2 or 4 days of training performed significantly better than DBA/2 mice, but their superiority disappeared during the second half of an 8 days-place training period. The pCREB levels of these mice were measured 30 min after place training and compared with those of mice that received only cued training. Changes in pCREB levels of C57BL/6 mice were greater in the hippocampal CA3, hippocampal dentate gyrus, orbitofrontal and medial PFC than those of DBA/2 mice, when mice of both received the switched place training for 2 days. We further investigated the roles of orbitofrontal and medial PFC among these brain regions showing strain differences, by destroying each region using selective neurotoxins. C57BL/6 mice with orbitofrontal lesions were slower to acquire the place learning and continued to use the cued search acquired during the cued training phase. These findings indicate that mouse orbitofrontal cortex (OFC) pCREB is associated with behavioral flexibility such as the ability to switch a learning task.

## Introduction

The prefrontal cortex (PFC) is known to be responsible for interplay between brain structures during switching from a memory system to the other (Ragozzino et al., 1999a,b; Rich and Shapiro, 2007, 2009; Bissonette et al., 2008). Rats with PFC inactivation have difficulty in switching to a new task (Rich and Shapiro, 2007). A study that recorded medial PFC neuronal activity during switching a learning task reported increases in activity when switching from a hippocampal-dependent place learning task to a dorsal-striatum-dependent cued learning task (Rich and Shapiro, 2009).

Further evidence for a role of the PFC in the switch between learning tasks come from a study of inbred strains of mice that show different behavioral performances in switching the learning task. C57BL/6 is an inbred strain commonly used to construct genetically modified mouse models, including models for neurological diseases such as Alzheimer’s disease (AD), and mice with conditional knockouts created using the Cre-LoxP recombination system (Ashe, 2001; Brooks et al., 2004; Mishina and Sakimura, 2007). C57BL/6 mice have been reported to show better performance in hippocampal-dependent tasks than DBA/2 mice (Paylor et al., 1994; Passino et al., 2002; Sung et al., 2008). Furthermore, C57BL/6 mice performed better than DBA/2 mice in a task that required switching between cued learning and place learning (Cho and Han, 2016). These behavioral differences were closely tied to strain differences in phosphorylated cAMP response element binding protein phosphorylated CREB (pCREB) levels in the PFC (Cho and Han, 2016). On the other hand, C57BL/6 mice with damage to the medial PFC and orbitofrontal cortex (OFC) showed impairments in attentional set shifting, an animal version of the Wisconsin Card Sorting task (Bissonette et al., 2008).

Prefrontal neuronal activity changed selectively when switching a learning task (Rich and Shapiro, 2009). C57BL/6 mice performed better in switching learning task and showed higher pCREB levels in the PFC, compared with DBA/2 mice (Cho and Han, 2016). However, when a switched learning task is adapted and stable with continued training, participation of PFC remains to be determined. Therefore, the present experiments were conducted to examine how long PFC is activated in switching the learning task from a cued to a place learning. To accomplish this, all mice received the cued training for 4 days in a water maze and then received three different durations of place training (2, 4, or 8 days). After completion of the place learning task, as a reference point of pCREB levels in mice of each strain that received only cued training, changes in pCREB levels induced by the switched place training were measured in the OFC and medial prefrontal cortex (mPFC). Changes in pCREB levels of the hippocampus and dorsal striatum were also measured.

C57BL/6 mice performed evidently better than DBA/2 mice when the switched place training was performed for 2 or 4 days. Strain difference disappeared during the second half of an 8 days-place training period. When changes in pCREB levels was measured after mice of both received the switched place training for 2 days, significant stain differences were found in the hippocampal CA3, hippocampal dentate gyrus, OFC, and mPFC. Notably, changes in pCREB levels of medial region of dorsal striatum, measured after day 8, were greater in C57BL/6 mice than DBA/2 mice.

A second experiment investigated the effects of OFC or mPFC lesions on the switch from cued to place learning, on the basis that the OFC and mPFC contribute differently to cognitive flexibility (Ragozzino, 2007) and that change in pCREB levels of OFC and mPFC was greater in C57BL/6 mice than DBA/2 mice after day 2. Sham-operated C57BL/6 mice learned to use the spatial search acquired during the place training phase, whereas C57BL/6 mice with OFC lesions did not switch to a spatial search, but rather continued to use the previously learned cued search.

## Materials and Methods

### Animals

Sixty-seven male C57BL/6 and 32 male DBA/2 mice (3 months old, specific pathogen free) were obtained from Charles River Co. (Gapeung, South Korea) at the beginning of the experiments. The mice were housed in groups of four animals to a cage, in a temperature- and humidity-controlled room, on a 12 h light/dark cycle (lights on, 07:00–19:00 h). Food and water were available *ad libitum*. All testing was performed during the light cycle. All experiments were conducted in accordance with the Konkuk University Council Directive for the use and care of laboratory animals, and NIH animal care guidelines. The Institutional Animal Care and Use Committee of Konkuk University approved all protocols described in this study.

### Surgery

C57BL/6 mice were randomly assigned to one of three groups for the lesion experiment: OFC lesion, mPFC lesion, and sham-operated controls. Under isoflurane inhalation anesthesia, bilateral stereotaxically targeted lesions were made in the mPFC [anteroposterior (AP), 1.9 mm; mediolateral (ML), 0.3 mm; ventral (V), 3.2 mm] or OFC regions (AP, 2.6 mm; ML, 1.2 mm; V, 2.8 mm) of mice, using established coordinates (Paxinos and Franklin, 2001). At each lesion site, approximately 0.25 μl of N-Methyl-D-aspartate (NMDA; 12.5 μg/μl) was injected using a 28-gauge cannula and Hamilton’s syringe. For the controls, autoclaved 0.01 M phosphate buffered saline (PBS) was injected into the mPFC and OFC. After surgery, all animals were given 2 weeks to recover and acclimate to daily handling.

### Apparatus

The water maze consisted of a circular tank (1.83 m diameter and 0.58 m height) with an escape platform (20 cm diameter) centered in one of the four maze quadrants. Water (26°C) was made opaque by the addition of nontoxic white paint. For cued training, the visible escape platform was raised 2 cm above the water surface. For place training, a hidden platform was located 0.5 cm beneath the surface. The maze was surrounded by white curtains (50 cm from the pool periphery), on which black felt patterns were affixed to provide three black extramaze (spatial) cues. These spatial cues were not placed adjacent to the hidden platform, so it was not possible for the mice to find the platform by swimming to a point lined up with a given cue. The water maze data were recorded using an HVS Image tracking system (Hampton, UK).

### Behavioral Training Procedure

Cued training: mice received 4 trials/day [with 10-min inter-trial intervals (ITIs), maximum trial duration of 60 s, with 20 s on the platform at the end of each trial], in which a visible platform was moved to a different location in the pool for each trial. Blank white curtains were drawn around the pool during cue training, to conceal any extramaze cues.

Place training: mice received 4 trials/day (10-min ITI, maximum trial duration of 60 s, with 20 s on the platform at the end of each trial), with each trial beginning with the mouse placed at one of four prespecified positions on the perimeter of the maze. The location of the platform remained constant across all training trials. The mice were placed into the water facing the wall and allowed to swim for a maximum of 60 s. A trial ended when the mouse climbed onto the available platform, or after 60 s had elapsed. If a mouse did not locate the platform during a trial, it was placed on the platform by the experimenter. The mice were left on the platform for 20 s, and then moved to a holding cage for a 10 min-ITI.

**Figure 1:**
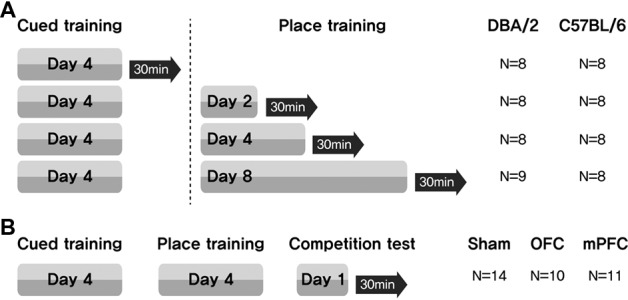
Experimental design for assessing the participation of brain structures related to a switch in learning task. **(A)** Mice received cued training for 4 days, followed by different durations of place learning. Black arrows indicate sacrifice time points after the place training, for phosphorylated CREB (pCREB) immunostaining. **(B)** Two weeks after surgery, three groups of mice received cued training for 4 days and then place training for 4 days. On the 9th day, they were given a competition test for two trials. N, number of animals used per group.

Learning task-switching for C57BL/6 and DBA/2 mice: C57BL/6 and DBA/2 mice first received cued training for 4 days. These mice were then assigned to one of four groups (Days = 0, no place training; Days = 2, place training for 2 days; Days = 4, place training for 4 days; Days = 8, place training for 8 days). Thirty minutes after the final training trial (on the 4th, 6th, 8th or 12th day of the full training protocol), the mice were sacrificed (Figure 1A).

Learning task-switching for C57BL/6 mice with lesions of the OFC or mPFC: all mice received cued training for 4 days, followed by place training for 4 days. On day 9, a competition test was given in which the visible platform was positioned in the northwest quadrant (opposite from the hidden platform position on the place training days), and the usual extramaze cues were displayed on the surrounding curtain. Two trials were conducted, with starting points equidistant from the two platform locations (NE and SW) (Figure 1B). Video recordings were analyzed to determine whether the mice swam at the previous hidden platform location prior to escaping to the visible platform (Figure 7E). Thirty minutes after the final test trial, the mice were sacrificed.

**Figure 2:**
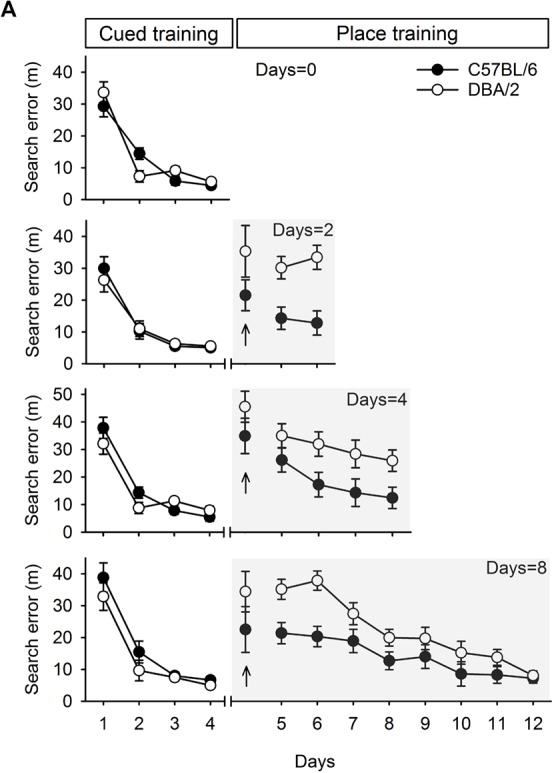
Performances of C57BL/6 and DBA/2 mice in the learning task required switching from cued to place learning. **(A)** C57BL/6 and DBA/2 mice received only cued training for 4 days. The other cohort of mice received cued training for 4 days and then place training for 2, 4, or 8 days. Differences between the C57BL/6 and DBA/2 mice were evident in the mice that received place training for 2 or 4 days. In the cohort that received 8 days of place training, the C57BL/6 mice performed better in the early phase of place learning than the DBA/2 mice, but no differences were observed in the late phase. No stain differences were found on the first trial in the first day of place training (upright arrow).

**Figure 3:**
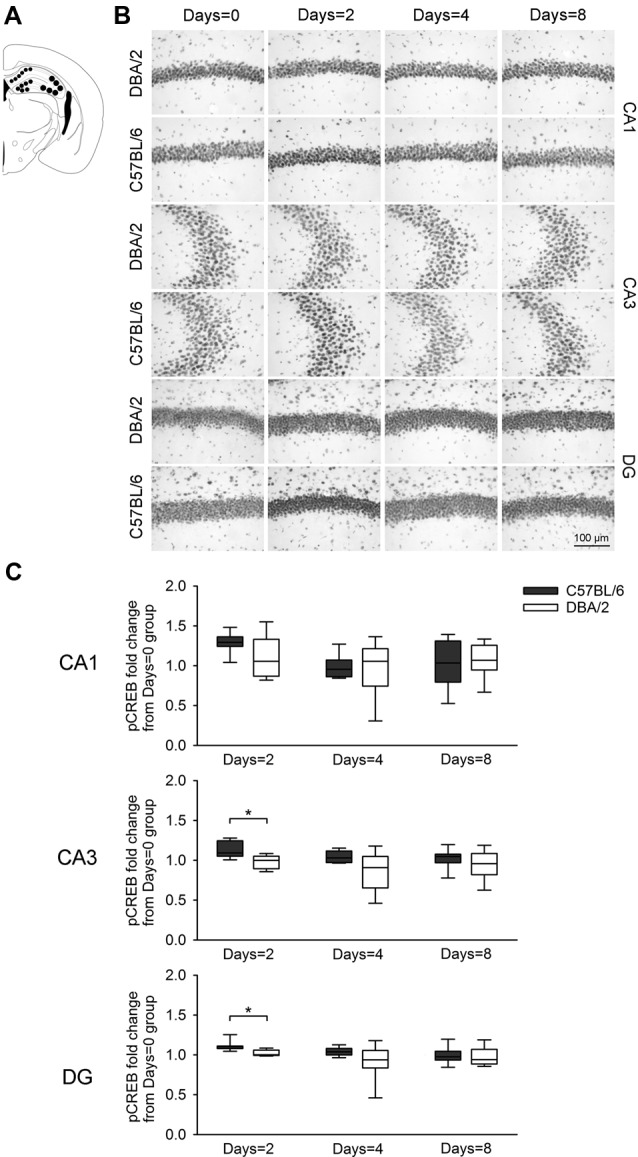
Changes of pCREB immunoreactivity in hippocampal regions Cornu Ammonis1 (CA1), CA3, and DG, 30 min after the learning task-switching from cued to place learning. **(A)** Immunostained cells with pCREB antibodies were measured within the marked areas (circle) of the CA1, CA3, and DG regions of the hippocampus. Photomicrographs **(B)** and quantification **(C)** of pCREB immunoreactivity in the hippocampal CA1, CA3, and DG regions of the C57BL/6 and DBA/2 mice. Levels of pCREB immunoreactivity were normalized with pCREB levels in mice of each strain that received only cued training (the Days = 0). Significant strain differences in pCREB levels of the CA3 and DG were found at Days = 2 groups that received the switched place training for 2 days (*). Scale bar = 100 μm. DG, dentate gyrus.

**Figure 4:**
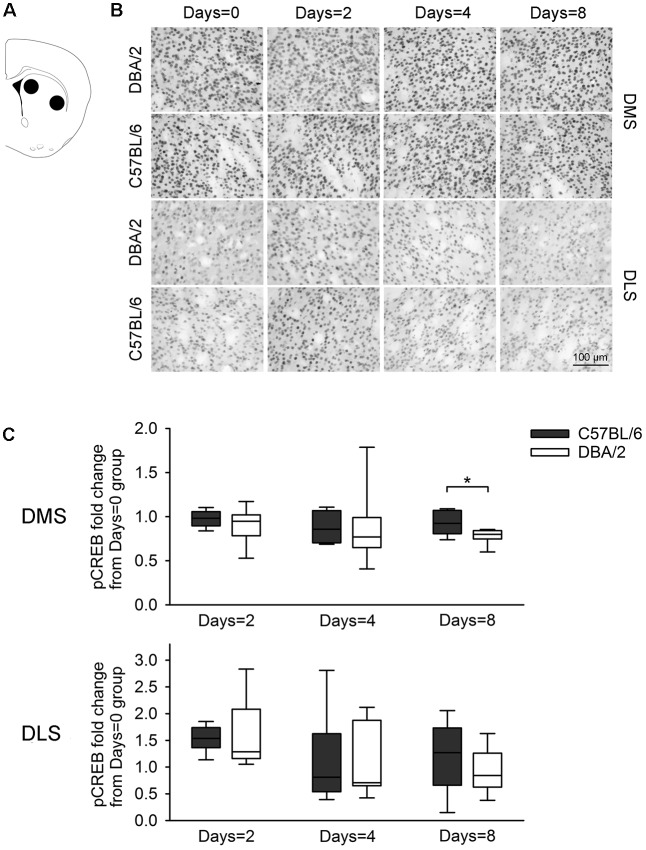
Changes of the pCREB immunoreactivity of dorsal striatal subregions, 30 min after the learning task-switching from cued to place learning. **(A)** Immunostained cells with pCREB antibodies were measured within the marked areas (circle) of the dorsomedial and dorsolateral regions of the striatum. Photomicrographs **(B)** and quantification **(C)** of pCREB immunoreactivity in the dorsomedial and dorsolateral striatum of C57BL/6 and DBA/2 mice. Levels of pCREB immunoreactivity were normalized with pCREB levels in mice of each strain that received only cued training (the Days = 0). Significant strain differences in pCREB levels of the DMS were found at Days = 8 groups that received the switched place training for 8 days (*). Scale bar = 100 μm. DMS, dorsomedial striatum; DLS, dorsolateral striatum.

**Figure 5:**
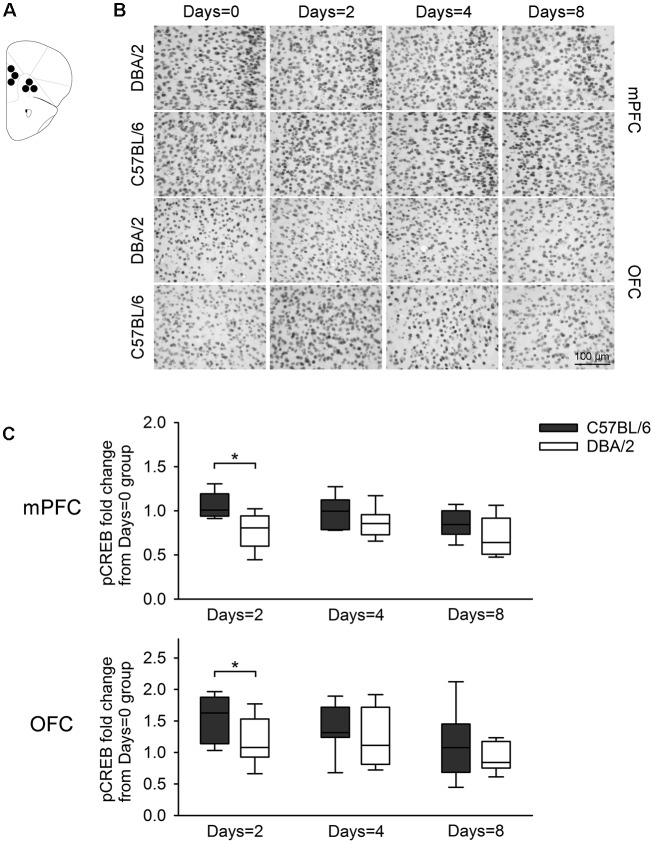
Changes of pCREB immunoreactivity in prefrontal subregions, 30 min after the learning task-switching from cued to place learning. **(A)** Immunostained cells with pCREB antibodies were measured within the marked areas (circle) of the mPFC and the OFC. Photomicrographs **(B)** and quantification **(C)** of pCREB immunoreactivity in mPFC and OFC of C57BL/6 and DBA/2 mice. Levels of pCREB immunoreactivity was normalized with pCREB levels in mice of each strain that received only cued training (the Days = 0). Significant strain differences in pCREB levels of the mPFC and OFC were found at Days = 2 groups that received the switched place training for 2 days (*). Scale bar = 100 μm. mPFC, medial prefrontal cortex; OFC, orbitofrontal cortex.

**Figure 6:**
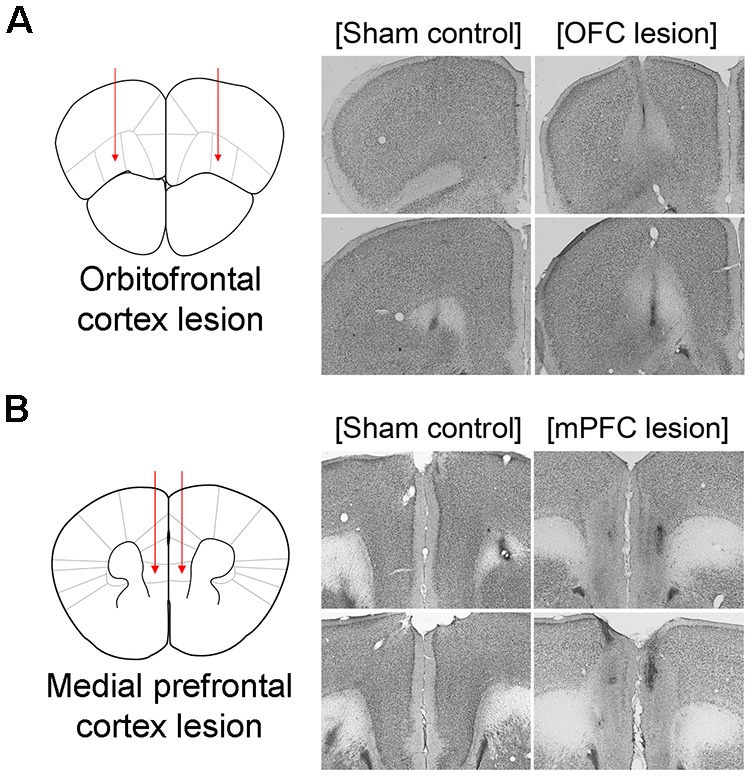
Schematic illustration of lesioned areas and representative photomicrographs of thionin-stained brain sections. *Left*, Schematic illustration of lesioned areas in OFC **(A)** and mPFC **(B)**. *Right*, Representative photomicrographs of thionin-stained brain sections from phosphate buffered saline (PBS)-injected sham control brains and brains with N-Methyl-D-aspartate (NMDA) Injections. OFC, orbitofrontal cortex; mPFC, medial prefrontal cortex.

**Figure 7:**
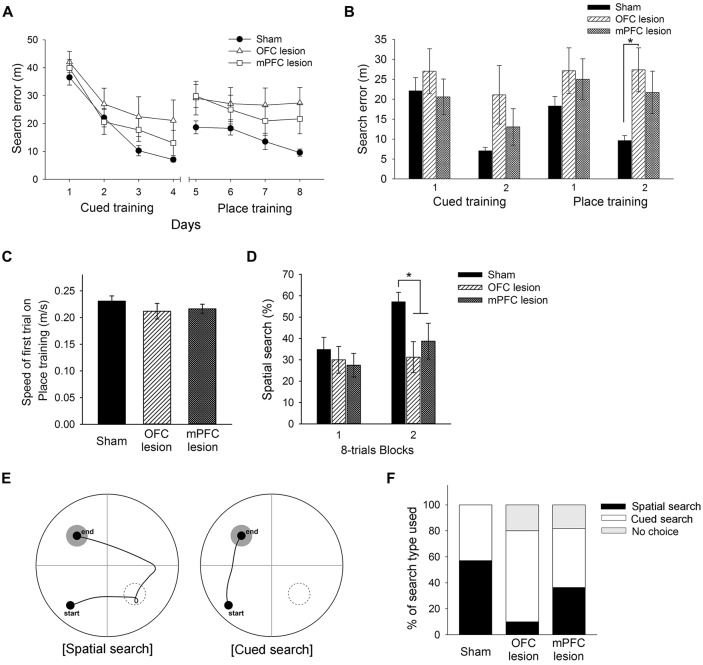
Performance of control mice, mice with OFC lesions, and mice with mPFC lesions in the learning task required the switch from cued to place learning. **(A)** Performances of control mice, mice with OFC lesions, and mice with mPFC in the cued training and the switched place training. Mice with OFC lesions showed significantly more search errors than control mice in the second half of the place training phase **(B)**. No differences between groups in swimming speed were found on the first trial of place training **(C)**. Mice with OFC or mPFC lesions used a spatial search less than control mice in the second half of the place training phase **(D)**. Representative swim paths are shown for a mouse using a “spatial search” and a mouse using a “cued search” in the competition test **(E)**. Mice with OFC lesions used the cued search rather than the spatial search **(F)**. The black rectangle indicates the percentage of mice that used the spatial search, whereas the white rectangle indicates the percentage that used the cued search. “No choice” (gray rectangle) indicates the percentage that not show any search type. control, sham-operated control mice; OFC, orbitofrontal cortex; mPFC, medial prefrontal cortex.

### Immunohistology

For pCREB measurements, 30 min after the final trial, the mice were injected with a ketamine-xylazine solution (1 ml/kg) and transcardially perfused with ice cold 0.1 M sodium phosphate buffer (pH 7.4), until all blood was removed. This was followed by perfusion with ice cold 4% paraformaldehyde in 0.1 M PBS. The brains were removed, post-fixed in 4% paraformaldehyde for 48 h, then transferred to 30% sucrose until they sank. Each brain was sliced into 45 μm coronal sections, from the olfactory bulb to the end of the hippocampus. Endogenous peroxidase activity was quenched by 30 min incubation of free-floating sections in 3% H_2_O_2_/10% MeOH in PBS. The sections were then incubated for 1 h at room temperature, in PBS with 0.3% Triton-X 100 containing 10% serum (PBS-T-S). Next, the sections were incubated with pCREB antibodies (1:2,000, Milipore, Billerica, MA, USA) for 17 h at 4°C, in PBS-T-S. The sections were then incubated for 1 h with goat anti-rabbit antibodies (1:1,000, Vector, Burlingame, CA, USA), and for another 1 h with ExtrAvidin peroxidase conjugate (1:1,000, Sigma Aldrich, St. Louis, MO, USA). Finally, the sections were reacted with a Vector SG substrate kit (Sigma Aldrich) for peroxidase, and mounted on resin-coated slides, after which they were dried for up to 5 days. The slides were then cover-slipped using Permount reagent.

For confirmation of neurotoxic lesions of the OFC and mPFC, brains were sectioned coronally using a freezing microtome, the sections were mounted on slides, and the slides were Nissl-stained.

### Image Acquisition and Analysis

The brain sections were imaged using a Nikon microscope (Tokyo, Japan) and a Progres^®^ camera (Harvard, Holliston, MA, USA) interfaced to a computer. This system was used in conjunction with Progres system software (Progres^®^ capture pro V2.8.8) for image acquisition. Quantitative analyses of pCREB-positive signals were performed using a computerized image processing system (Metavue, MD, USA) coupled to an optical microscope. The quantification of pCREB-positive signals was carried out at 100× magnification for the hippocampus, and at 40× magnification for the dorsal striatum and PFC. Structures were defined according to the Franklin and Paxinos atlas (Paxinos and Franklin, 2001). The pCREB-positive signals were counted bilaterally in at least six sections per animal, spaced 150 μm apart. The pCREB-positive signals were quantified in the following regions of interest: subfields *Cornu Ammonis*1 (CA1), CA3, and dentate gryrus (DG) of the hippocampus, dorsomedial and dorsolateral parts of the striatum, OFC, and mPFC. The area of pCREB-positive signals in each structure in each animal was divided by the mean area of those in the corresponding structures in the control mice (which received only 4 days of cued training), to generate a normalized measure for each animal.

### Behavioral and Statistical Analyses

During each trial, in the cued and place training phases, the distance of the mouse from the escape platform was sampled 10 times per second, and these values were averaged in 1 s bins. The cumulative search error was then calculated as the sum of the 1 s averages, corrected for the start location for each trial. Search errors in the cued and place training phases were analyzed separately, using two-way repeated measures analysis of variance [analyses of variance (ANOVA); group × trial session (day)], to evaluate differences between C57BL/6 and DBA/2 mice, or between mice with OFC lesions, mice with mPFC lesions, and control mice. *Post hoc* analyses (least significant difference) were conducted to examine strain and group differences. The swimming search patterns were classified either as spatial search or non-spatial search, for each mouse, on every trial in the place training phase, according to the criteria adopted by Janus (2004). For the lesion study, the swimming speeds during the first trial of place training were analyzed using a one-way ANOVA. Data were expressed as mean ± SEM, in the analyses that parametric statistics was used. Because homogeneity of variance in pCREB changes by the duration of the switched place training was violated, we used Mann-Whitney *U* test, a distribution-free statistic, and data was expressed as the box-plot.

For the competition test, the mice were given two trials. Figure 7E shows representative swim paths for mice using two different search types. Mice designated as using a “spatial search” visited the location where the platform had been on the previous training days, before escaping to the newly located visible platform. In contrast, mice using a “cued search” swam directly to the visible platform in its new visible platform location (McDonald and White, 1994). Using the criteria established by McDonald and White (1994), the mice were classified as using a spatial search if they visited the previous platform location during either of the two trials. All other mice were classified as using a cued search. A Chi-square test was used to evaluate differences in the frequency of search types during the competition test. *P* values less than 0.05 were considered significant.

## Results

### Learning Task-Switching From Cued to Place Learning

Performance at the switching task from cued training (visible platform) to place training (hidden platform), is shown in Figure 2. Consistent with earlier reports (Cho and Han, 2016), both C57BL/6 and DBA/2 mice in the four groups learned to escape with comparable proficiency in the cued training phase (*F*_(1,15)_ < 3.44, *p* > 0.08), as measured by the reductions in search errors. These mice improved over the course of cued training, as indicated by a significant effect of training (*F*_(3,45)_ > 43.87, *p* < 0.001). No significant interaction between strain and training was found in any group except the Days = 0 group (*F*_(3,42)_ = 4.13, *p* < 0.05). After the switch to place training, both C57BL/6 and DBA/2 mice did not show differences on the first trial in the first day of place training (Days = 2, *t*_(14)_ = 1.46, *p* = 0.17;Days = 4, *t*_(14)_ = 1.24, *p* = 0.24; Days = 8, *t*_(15)_ = 1.24, *p* = 0.23) and progressively learned to escape to a hidden platform. However, search errors for the C57BL/6 mice decreased rapidly over the course of training, whereas the DBA/2 mice performed comparatively poorly in the early days of place learning. Specifically, a two-way repeated measures ANOVA of place learning over 2 days (the Days = 2 group) showed a significant effect of strain (*F*_(1,14)_ = 22.44, *p* < 0.001), but no effect of training (*F*_(1,14)_ = 0.07, *p* = 0.80) or interaction between strain and training (*F*_(1,14)_ = 0.47, *p* = 0.50). Statistical analysis of 4 days of place learning (the Days = 4 group) showed that the mean effects of strain and training were both significant (*F*_(1,14)_ = 7.51, *p* < 0.05; *F*_(3,42)_ = 4.16, *p* < 0.05), but there was no significant interaction between strain and training (*F*_(3,42)_ = 0.30, *p* = 0.82). Statistical analysis of 8 days of place learning (the Days = 8 group) showed that the effects of strain and training were both significant (*F*_(1,15)_ = 10.15, *p* < 0.001; *F*_(7,105)_ = 17.83, *p* < 0.001), but again there was no interaction between strain and training (*F*_(7,105)_ = 1.86, *p* = 0.08). However, the DBA/2 mice did finally learn to find the hidden platform in the late phase of place training, from session 9–12. Subsequent *post hoc* analyses showed that the strain differences were evident until day 3 (*p* < 0.05), but, afterwards disappeared. The two strains of mice showed no differences in swimming speed (*t*_(47)_ = -0.11, *p* = 0.91).

### Changes of pCREB Levels in the Hippocampus, Dorsal Striatum, and Prefrontal Cortex Induced by the Switched Place Training

We reported previously that strain differences in hippocampal and prefrontal pCREB levels, measured after completing a task that involved the switch from cued (4 days) to place training (4 days), were closely tied to behavioral differences (Cho and Han, 2016). Therefore, the present experiment was conducted to measure changes of pCREB levels, in C57BL/6 and DBA/2 mice, of brain structures necessary for performing the switch of the learning task. Because the participation of these brain structures depended on the amount of training, the present experiment examined pCREB levels by measuring immunoreactivity in the hippocampus (CA1, CA3, and DG), dorsal striatum (medial and lateral), and PFC (medial and orbitofrontal) in C57BL/6 and DBA/2 mice with different durations of the switched place training. These mice were sacrificed either 30 min after the final cued training session on day 4 (the Days = 0 group, no place training), or after the final place training session on day 2, 4, or 8 (the Days = 2, Days = 4, and Days = 8 groups). For the examination of pCREB changes, the areas of pCREB-positive signals of brain structures from the mice that received place training (Days = 2, 4, 8) were normalized with respect to those of the mice of the same strain that received only cued training (Days = 0).

Figure 3 shows changes of pCREB levels in the hippocampal subregions by the degree of the switched place training. Mann-Whitney *U* test revealed significant strain differences in CA3 and DG pCREB changes at Days = 2 groups (*U*s < 8, *p* < 0.05), but no strain differences were observed in CA1 pCREB changes on any groups with different training durations (*U*s > 16) and in CA3 and DG pCREB changes at Day = 4 and Days = 8 groups (*U*s > 17). Figure 4 shows changes of pCREB in dorsal striatum subregions by the degree of the switched place training. Statistical analysis on pCREB changes of dorsomedial striatum revealed strain difference on Days = 8 (*U* = 12, *p* = 0.04). No differences between stains were found at any other groups and any subregions (*U*s > 23). Figure 5 shows changes of pCREB in PFC subregions by the degree of the switched place training. Statistical analyses showed strain differences in mPFC and OFC pCREB changes at Days = 2 groups (*U*s < 14, *p* < 0.05). No strain differences were observed at Days = 4 and Days = 8 groups (*U*s > 14).

### Effects of OFC or mPFC Lesions on Switched Place Learning and Selection of Search Type in the Switching Task From Cued to Place Learning

Based on the results above, the present experiment examined the effects of OFC or mPFC lesions on the switching of the learning task. Thionin staining of the PFC showed extensive damage to the OFC or mPFC in the lesioned C57BL/6 mice (Figure 6). Figure 7A shows the performances of mice subjected to the learning task-switching. A two-way repeated measures ANOVA revealed that all mice, regardless of lesions, progressively learned to find a visible platform, as indicated by the decreasing levels of search errors from Day 1 to Day 4 (*F*_(3,96)_ = 58.84, *p* < 0.001), and that no significant effect of group (*F*_(2,32)_ = 1.78, *p* = 0.19) and no group × day interaction (*F*_(6,96)_ = 1.13, *p* = 0.35), were found. Statistical analyses of performance in the subsequent place training, from day 5 to day 8, showed a significant effect of training (*F*_(3,96)_ = 6.34, *p* < 0.001), but no significant effect of group (*F*_(2,32)_ = 2.94, *p* = 0.07), and no group × day interaction (*F*_(6,96)_ = 1.35, *p* = 0.24). In addition, we divided the data into two blocks (the first 8 trials vs. the second 8 trials) for the cued training and the place training, to examine group differences in the development of cued and place learning. A one-way ANOVA of the cued training data revealed no significant group effect for the first or second block (*F*_(2,32)_ = 1.88, *p* = 0.17; *F*_(2,32)_ = 2.83, *p* = 0.07). A one-way ANOVA of the place training data revealed no significant group effect for the first block (*F*_(2,32)_ = 2.08, *p* = 0.14), but a significant group effect for the second block (*F*_(2,32)_ = 3.42, *p* < 0.05). According to subsequent *post hoc* analyses, the mice with OFC lesions showed more search errors in the second block of place training than the control mice (*p* < 0.05; Figure 7B). On the first trial of place training (Figure 7C), however, performance was equivalent across the groups, indicating that the patterns of random search were similar in control mice and mice with lesions (*F*_(2,32)_ = 0.84, *p* = 0.44). The search types adopted by the mice during place training were analyzed using criteria reported earlier (Janus, 2004). A one-way ANOVA of the spatial search adopted revealed no significant effect of group for the first block (*F*_(2,32)_ = 0.74, *p* = 0.49), but a significant effect for the second block (*F*_(2,32)_ = 4.71, *p* < 0.05). *Post hoc* analyses showed that the control mice developed a spatial search during the spatial training, but mice with OFC or mPFC lesions did not (Figure 7D). In the competition test (Day 9), 8 of 14 control mice adapted a spatial search, whereas only 1 of 10 OFC lesion mice did (*χ*^2^ = 7.05, *p* < 0.05). On the other hand, 4 of 11 mice with mPFC lesions adapted a spatial search, which was not significantly different from the control mice (Figures 7E,F).

## Discussion

The present study examined involvements of brain structures associated with switching of learning task, by measuring changed levels of pCREB, along with behavioral differences between C57BL/6 and DBA/2 mice. Consistent with our earlier report (Cho and Han, 2016), the two inbred mouse strains displayed similar performance in the cued training, but considerable strain differences were observed in mice that received the switched place learning for 2 or 4 days. The strain differences were observed in the early phase, but not in the later phase in mice that received the switched place learning for 8 days. That is, relative to C57BL/6 mice, DBA/2 mice were slower to learn to find the hidden platform but improved their performance in the late phase of the switched place learning.

pCREB has been used in rodents as a marker of memory formation after task-specific learning (Colombo et al., 2003; Sung et al., 2008). In a previous study, a large behavioral difference between two strains of inbred mice was found at the task requiring a switch from cued to place learning, and strain differences in pCREB levels were evident in the hippocampus and PFC (Cho and Han, 2016). The present study also found that changes in hippocampal and prefrontal pCREB levels were greater in C57BL/6 mice than DBA2/mice at Days = 2 group that received the switched place training for 2 days. Specifically, changes in pCREB levels were greater not only in hippocampal CA3 and DG, but also in mPFC and OFC. Notably, pCREB levels in dorsomedial striatum showed strain differences at Days = 8 that received the switched place training for 8 days.

These results could be explained by the hypothesis that the PFC, along with the hippocampus, is one of the brain structures necessary for spatial learning (Colombo et al., 2003; Churchwell et al., 2010; Baudonnat et al., 2011). For example, rats with infusions of lidocaine into the mPFC and CA1 showed impairments of encoding and retrieval of spatial memory (Churchwell et al., 2010). PFC pCREB levels were increased after acquisition of a spatial learning task with food reward (Baudonnat et al., 2011).

On the other hand, the hippocampus and PFC are also involved in the learning task-switching. Acetylcholine release in the hippocampus was increased in the early phase of a task that required switching from place to cued learning (Chang and Gold, 2003). A series of studies has shown that the PFC is critical for a switch between spatially guided responses and egocentric responses (Ragozzino et al., 1999a,b). In the present task, after receiving the switched place training for 2 days, changes in pCREB levels of mPFC and OFC were greater in C57BL/6 mice than DBA/2 mice. The same result was obtained from hippocampal CA3 and DG, which is consistent with the earlier reports that adult neurogenesis in CA3 was involved in the flexible use of spatial learning strategy (Garthe et al., 2009) and that Colchicine lesion of DG impaired flexibility of use of environmental cues in the spatial task in the water maze (Xavier et al., 1999). Interestingly, in our earlier report that, at the switching of the learning task (from cued to place learning or vice versa), C57BL/6 mice showed better performance both at cued learning following place learning and at place training learning following cued learning. In both switching of learning task, higher hippocampal CREB activation was observed in C57BL/6 mice than DBA/2 mice (Cho and Han, 2016). These reports suggest that CREB signaling in the hippocampus and PFC might be recruited at the early stage of the switch of learning task.

In addition, it has been reported that the dorsomedial striatum is related to goal-directed learning, and the dorsolateral striatum is related to response learning (Yin and Knowlton, 2006). Especially, a number of study has shown that the dorsomedial striatum is involved in the spatial learning and reversal learning (Pisa and Cyr, 1990; Devan and White, 1999) and that the linkage between dorsomedial striatum and PFC is related to behavioral flexibility (Groenewegen et al., 1990; Groenewegen and Berendse, 1994). For example, the inactivation of the dorsomedial striatum impaired shift learning between response and visual cue discrimination (Ragozzino et al., 2002). Our previous study also reported that pCREB levels in the dorsomedial striatum were related to behavioral differences between C57BL/6 and DBA/2 mice, in the switch from place to cued (Cho and Han, 2016). Furthermore, in the present study, changes in pCREB levels of dorsomedial striatum were greater in C57BL/6 mice than DBA/2 mice at Days = 8 groups that received the switched place training for 8 days.

The study investigating the spatial-temporal dynamics of pCREB of mice with spatial reference training in the water reported direction differences (i.e., increase in the hippocampus vs. decrease in the striatum), regional differences (CA1 vs. other brain regions), and dynamics (biphasic vs. monophasic; Mizuno et al., 2002; Porte et al., 2008). However, the present study measured pCREB levels at one time point after the switched spatial training. Hence, to extend and confirm the present results, further study to measure pCREB levels at various time points after the task is required.

The association of the PFC with switching of learning task has been studied using lesions or inactivation of the PFC in rats. For example, rats with mPFC inactivation by tetracaine, a local anesthetic, failed to shift from hippocampal to striatal-dependent tasks, or vice versa (i.e., extradimensional shift), but did not impair reversal learning (i.e., intradimensional shift), in a cross-maze (Ragozzino et al., 1999a). On the other hand, OFC damage impaired reversal learning (Kim and Ragozzino, 2005), but did not impair performance in an extradimensional shift task (McAlonan and Brown, 2003). Based on these reports and the results of the present study, we examined the effects of lesions of prefrontal subregions (OFC and mPFC) on switches of learning task. As mPFC or OFC damage did not affect acquisition of cued or place training in the studies described above, C57BL/6 mice with OFC or mPFC lesions showed intact acquisition of cued training. In the place training that followed the cued training, mice with OFC lesions or mPFC lesions used less spatial search during the second half of the switched spatial training session, compared with those of control mice. But, from several behavioral measurements, mice with OFC lesions had more difficulty in switching to a search type appropriate for the new training, as compared to control mice and mice with mPFC lesions.

Though it is well known that OFC and mPFC are functionally distinct (Ragozzino, 2007), the present pCREB results indicated that, rather than one region being responsible for switching the learning task in the water maze, several regions were involved. In addition, the present lesion experiment suggests that, though OFC and mPFC are necessary for the present switching task, OFC play a more central role. On the other hand, it is also reported that rats with lesions of PFC were impaired in the acquisition and transfer of a general maze-skill, not loss of maze-specific information (Winocur and Moscovitch, 1990). Therefore, the present results can be interpreted that the mice with OFC and mPFC were slower to acquire the switched spatial learning due to no transfer of the general maze-skill that would be learned in the cued learning.

Non-associative effects exposed to the subject during cued training might in part contribute behavioral strain differences observed in the present switching task from cued to place training in water maze. Therefore, further investigation is needed to determine non-associative effects or pre-exposure effects of the water maze setting in the cued training on the subsequent place training. Especially, the yoked subject as a non-associative control would have the same exposure to swimming in the absence of the platform and to time on the platform that a counterpart would exhibit in the cued training.

In summary, as is evident from previous reports, the hippocampus and striatum are important neural substrates for place and cued training (Morris et al., 1982; Yin and Knowlton, 2006), and the PFC contributes to switching between two tasks (Ragozzino et al., 1999a). The present study assessed time of participation of the hippocampus, striatum, and PFC in switching from cued to place learning by determining levels of pCREB after the switched place learning with different training durations. The results indicate that the OFC in conjunction with mPFC, hippocampal CA3, and DG participate in early stage of the switching from the one task to another. These findings, and the switching of the learning task used in the present study, might be useful for studying the cognitive functions of the PFC in AD model transgenic mice, or in mice with conditional gene targeting using Cre-LoxP recombination (Mishina and Sakimura, 2007; Cho et al., 2014).

## Author Contributions

WHC and JSH conceived and designed the experiments. WHC and JCP performed the experiments. WHC, WJ, JC and JSH analyzed and discussed the data and wrote the manuscript.

## Conflict of Interest Statement

The authors declare that the research was conducted in the absence of any commercial or financial relationships that could be construed as a potential conflict of interest.
